# Lipoma in Parotid Gland: A Rare Case Report

**DOI:** 10.1055/s-0042-1758795

**Published:** 2023-01-04

**Authors:** Sergio Santoso, Andra Rizqiawan, Indra Mulyawan, Asdi Wihandono, Muhammad Subhan Amir

**Affiliations:** 1 Oral and Maxillofacial Surgery Specialist Program, Faculty of Dental Medicine, Airlangga University, Surabaya, Indonesia; 2 Department of Oral and Maxillofacial Surgery, Faculty of Dental Medicine, Airlangga University, Surabaya, Indonesia; 3 Oncology Division, Department of Surgery, Faculty of Medicine, Airlangga University, Surabaya, Indonesia

**Keywords:** lipoma, parotid gland, parotidectomy, medicine, dentistry

## Abstract

Lipoma is a benign, rare, mesenchymal tumor found in the head and neck region, especially in the parotid gland. It thus requires a careful and precise examination to establish a diagnosis. A surgical procedure of the parotid gland is challenging due to the associated risk factor of facial nerve injury. We report a rare case of head and neck region lipoma between the superficial and deep lobe of the parotid gland. A 44-year-old female patient was presented with the chief complaint of a painless lump on the left front ear to the left cheek for about 1 year. There were no complaints of tooth pain before the lump appeared, and there were no lumps in other regions. A fine-needle aspiration biopsy, ultrasonography, and magnetic resonance imaging were all performed to establish the preoperative diagnosis and to plan the correct surgical approach. Lipoma was the initial clinical diagnosis, and a surgical excision with superficial parotidectomy and facialis nerve preservation was performed. Follow-up examinations were conducted to assess any facial nerve injury complications. Conclusion Lipoma rarely grows in the parotid gland. Careful diagnosis should be performed to establish a precise surgery for parotid dissection and facial nerve preservation.

## Introduction

Lipoma is a benign mesenchymal tumor that can be found anywhere in the body where fat is normally present, but it is rarely found in the head and neck region, even less so in the parotid region. 1 Only 0.1 to 0.5% of benign tumors and 25% of all lipomas and its variations are found in the head and neck region. 2 Lipoma of the parotid gland is uncommon, having a 4.4% average incidence across all parotid malignancies. 3 Lipomas are reportedly detected in the major salivary glands, buccal mucosa, lip, tongue, palate, vestibule, and mouth floor in the head and neck areas. 4 Salivary gland tumors are rare and represent for 2.5 to 6.5% of all cases, in which parotid gland is the most frequent site. 5 6 7 Males are 10 times more likely than females to have lipomas, which are often discovered between the fifth and sixth decades of life. 8 9 Most lipomas grow quietly, painlessly, and asymptomatically, and they may affect surrounding structures. 10

Due to the rarity of lipoma in the parotid gland regions, an accurate diagnostic method is required to make a conclusive diagnosis. A more accurate diagnosis of these tumors have been made possible by improved preoperative evaluation tools including magnetic resonance imaging (MRI) and fine-needle aspiration biopsy (FNAB). 1 9 A surgical procedure of these tumors is challenging because of the anatomical barrier where facial nerves cross throughout the parotid gland, thus increasing the risk of nerve injury and damage during surgery. A well-established surgical technique, special precautions, and attention to anatomical structures around the surgical area, as well as follow-up after surgery, are important to reduce and manage those risks. 8 This case report presents the diagnostic method and surgical approach of a rare case of head and neck region lipoma in the parotid region.

## Case Report

**Fig. 1:**
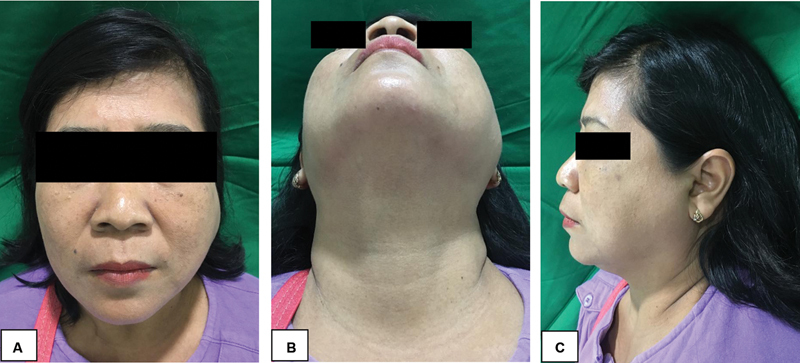
Patient's preoperative view showing swelling of the left parotid region. ( **A** ) Frontal extraoral view, ( **B** ) bottom extraoral view, and ( **C** ) left extraoral view.

A 44-year-old female patient with a lump on the left front ear to the left cheek for 1 year was presented to our department. The swelling was painless and had progressively increased during the previous year. There were no complaints of tooth pain, and no lumps were found in other regions. A physical examination showed a solitary mass on the left preauricular region, with a size within 4 × 3 × 2 cm, firm in consistency, and not compressible on palpation. The skin over the swelling appeared normal. There were no other head and neck masses that could be felt ( Fig. 1 ).

The patient's past medical history and psychosocial data presented no related information with the swelling. There was no family history of tumors, especially in the head and neck regions. An examination and therapy related to the swelling had never been performed on the patient. FNAB, ultrasonography (USG), and MRI were performed sequentially to establish a preoperative diagnosis and to plan the best surgical approach.

**Fig. 2:**
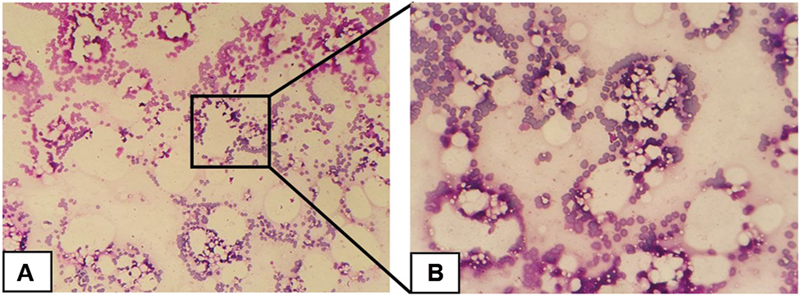
Fine-needle aspiration biopsy (FNAB) histopathology view (hematoxylin and eosin staining). FNAB results show distribution of acini gland epithelial cells with round-oval nuclei, fine chromatin, extensive cytoplasm, and distribution of erythrocytes, some lymphocyte inflammatory cells, and fat droplets. ( **A** ) 10× magnification and ( **B** ) 40× magnification.

**Fig. 3:**
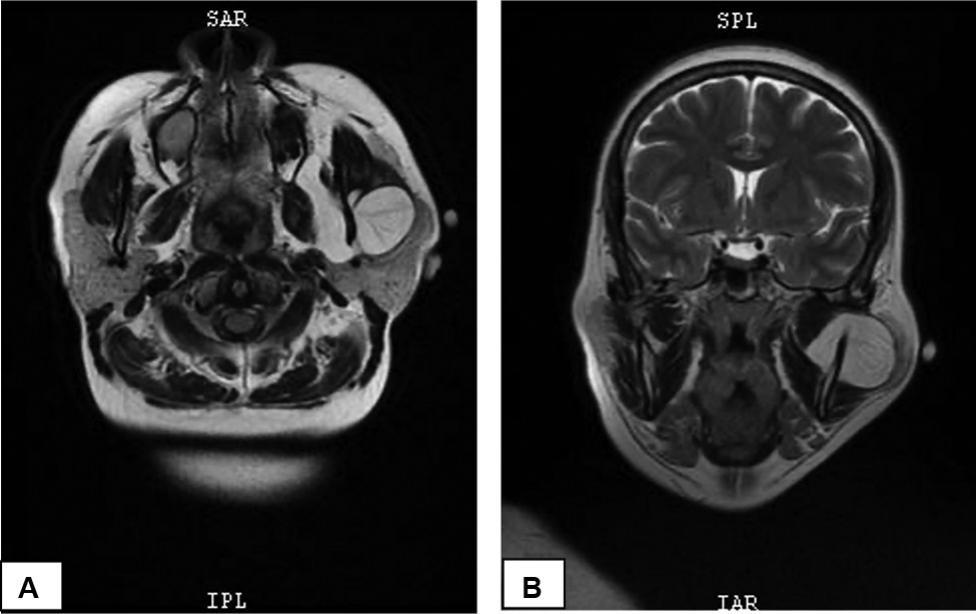
Preoperative T1-weighted magnetic resonance imaging (MRI) showed fat density noted in the left parotid gland. ( **A** ) Axial view and ( **B** ) coronal view.

The FNAB report revealed a distribution of epithelial cells with round-oval nuclei, fine chromatin, broad cytoplasm, and eosinophilic color with the background consisting of a distribution of erythrocyte cells, a few inflammatory lymphocyte cells, and fat droplets. The report also showed that there were no malignant cells, and it was suspected that the swelling was a benign lesion mass suggestive of lipoma ( Fig. 2 ). An USG examination revealed a solid mass in the left parotid with sharp and flat margins, a slightly hypoechoic mass with a size of 3.31 × 1.44 cm, and an enlarged left retroauricular lymph node with a size of 2.04 × 0.74 cm. The MRI showed a mass on the left parotid region with the intensity of fat growing from the medial of mandible coming out under the skin, with a firm boundary, flat edge, and a size of 4.21 × 3.38 cm located in the deep and superficial lobe of parotid gland, which suggested a parotid gland lipoma ( Fig. 3 ). Due to the rarity of lipomas in the head and neck region, the diagnosis in this case was difficult. Since USG examination in this instance cannot distinguish lipomas between the superficial and deep lobes of the parotid gland, an MRI examination should be performed to determine the precise location of the lipoma.

**Fig. 4:**
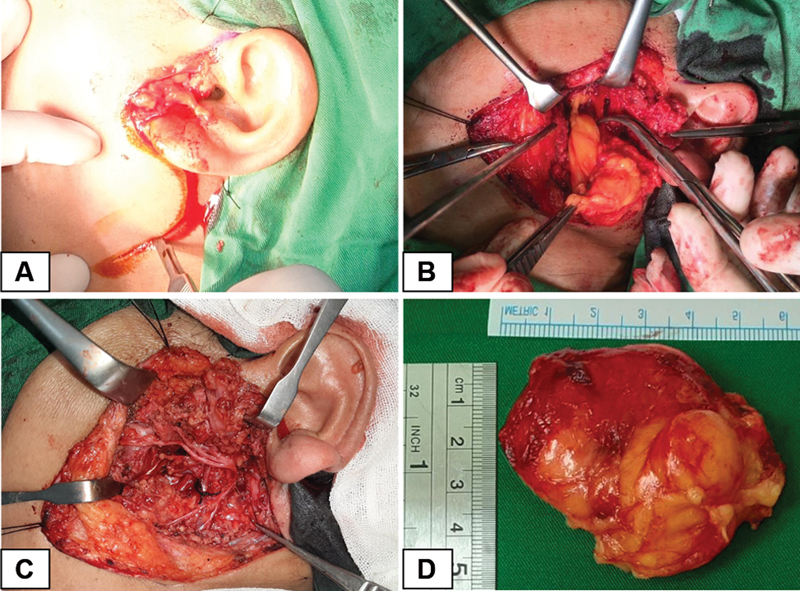
Intraoperative view: ( **A** ) Surgical approach using a classic Blair's incision. ( **B** ) The fatty yellowish mass comes out from the surrounding parotid tissue after blunt dissection. ( **C** ) Superficial parotidectomy and blunt dissection with facial nerve preservation. ( **D** ) Removed specimen. Lipoma with size 5.5 cm × 5 cm, well-circumscribed, soft, yellow, fatty mass covered with a thin fibrous capsule.

**Fig. 5:**
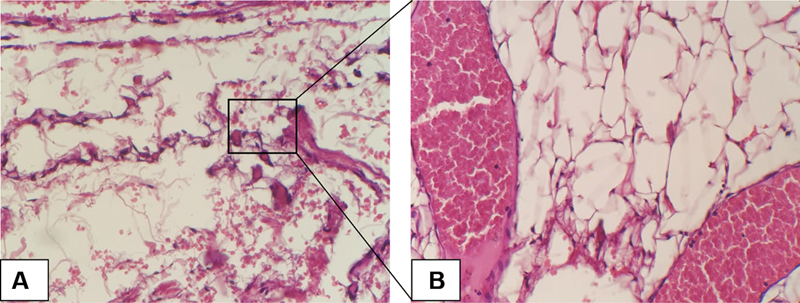
Histopathology view (hematoxylin and eosin staining). Postoperative histological diagnosis was lipoma characterized by a well-circumscribed tumor with fibrous capsule composed of lobules of uniform mature, univacuolated adipocytes rimmed by salivary gland parenchyma. ( **A** ) 10× magnification and ( **B** ) 40× magnification.

Using the classic Blair's incision, a surgical lipoma excision, facial nerve dissection, and superficial parotidectomy were performed ( Fig. 4A ). During surgery, a large mass was discovered close to the facial nerve in the left parotid area. Thus, superficial parotidectomy and blunt dissection were performed using hemostatic forceps (Aesculap, Germany) to preserve the main trunk of the facial nerve ( Fig. 4B ). The facial nerve main trunks were separated from the overlying superficial parotid lobe, revealing a yellowish fatty capsulated mass ( Fig. 4C ). Without causing significant damage to any neurovascular structures, the tumor was removed. The tumor was made up of a thin, fibrous capsule covering a well-defined, soft, yellow, fatty mass ( Fig. 4D ). A postoperative histopathological examination showed homogeneous mature adipocytes (round-oval nuclei), fine chromatin, and broad cytoplasm. A final diagnosis of parotid gland lipoma was obtained based on the preceding microscopic characteristics ( Fig. 5 ).

**Fig. 6:**
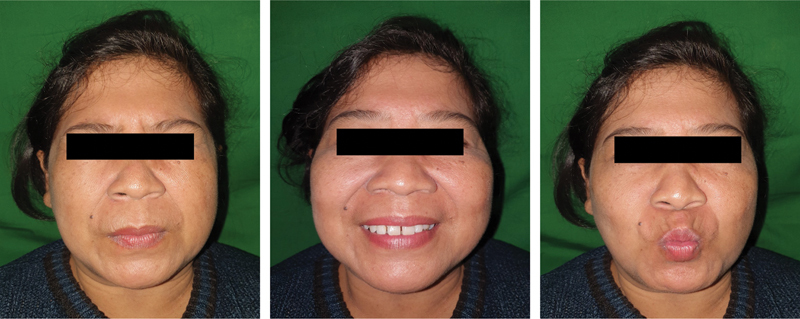
Patient facial nerve function evaluation 2 years after surgery is normal.

During the hospital stay, the patient was given antibiotics to prevent postoperative infection, nonsteroidal anti-inflammatory drugs, and corticosteroid anti-inflammatory drugs to reduce postoperative inflammation. The patient was discharged 3 days after the operation with a good prognosis assessment. Follow-ups were performed 3 months, 6 months, 1 year, and 2 years after the operation to observe any disease recurrence and facial nerve functions. There was no recurrence during the follow-ups. Facial nerve function was checked clinically by asking the patient to wrinkle the forehead to observe any asymmetry, close the eyes tightly to observe lid closure competence, smile to observe asymmetry of the nasolabial fold, and contract the mouth to observe orbicularis oris functions. There was no facial nerve dysfunction found during the examination ( Fig. 6 ).

## Discussion

The most common benign mesenchymal encapsulated tumors that resemble mature adipose tissue histologically are lipomas. Adipose tissue is known to exist in salivary glands; however, lipomas, particularly those of the parotid gland, are quite uncommon. 8 9 Lipomatous lesions of the parotid gland are considered as an initial diagnosis in parotid gland swelling. 2 Only 15 to 25% of lipomas and their variants are seen in the head and neck region on average, especially in the superficial and subcutaneous layers. 1 2 8 The reported incidence of lipoma varies by 4.4% among parotid tumors. 2 3 11 Males are more likely than females to develop parotid gland lipomas, which are most common in people's fifth to sixth decades of life (male-to-female ratio of 3:1). 1 8 12 The parotid lipoma presents clinically as a soft, slowly expanding, painless, well-defined, asymptomatic mass. Clinical diagnosis is generally challenging, likely as a result of the low clinical suspicion index. 1 2 8 In this case, lipoma was diagnosed on a 44-year-old female patient (fifth decade) on the left parotid region.

Numerous etiological factors were explored, such as inherited factors, trauma, diabetes, endocrine problems, corticosteroids, obesity, and radiation. 9 The most frequent cause of hematoma, lymphatic effusion, and fat necrosis, which leads in lipoma formation, is trauma, according to the clinical history. 1 12 Additionally, the majority of individuals only have a single lesion and do not have any risk factors. 1 In this case, the swelling was painless and progressively grew in size over 1 year.

Despite having a histological resemblance to mature adipose tissue, a fibrous capsule helps to identify lipoma from simple, normal fat aggregation. 1 Due to the rarity and the low clinical suspicion index of lipomas, the clinical diagnoses are typically challenging and the procedure performed must be precise to establish a definitive diagnosis. Hence, a diagnosis of the parotid lipoma is challenging, and an FNAB accompanied by an MRI determines its diagnosis preoperatively. 9 In this case, an FNAB, USG, and MRI were performed to establish a preoperative diagnosis and to plan the best surgical approach.

Nowadays, a histopathological examination and preoperative imaging play crucial roles in correctly diagnosing the nature and location of lesions. Their primary purpose is to remove extraparotid subcutaneous lesions, and second objective is to confirm the lipoma diagnosis. 1 9 The availability of USG, MRI, and FNAB examinations are needed to provide an accurate assessment of these tumors and a rational approach to the management of such cases.

An essential diagnostic technique for identifying parotid tumors is a FNAB. However, its accuracy is only approximately 50% in most parotid tumor cases. 9 An FNAB of a lipoma is often reported to have lobulated adipose tissue with thin capillaries traversing the lobules and thick walls. On the other hand, an FNAB is highly accurate for the diagnosis of lipomatous tumors, with 96% accuracy for lipomas. 2 In this case, the FNAB revealed no malignant cells, and they were suspected to be a benign lesion mass of preauricular sinistra, suggestive of lipoma. However, a frequently used FNAB in the diagnostic tool for a parotid mass does not offer enough data to establish a diagnosis. 13 14 Preoperative imaging such as USG and MRI are required to ensure perioperative diagnosis, as in this case.

Preoperative imaging for a case of lipoma usually comprises either a USG or MRI scan. 2 15 A USG is an affordable, nonionizing modality that can be used as an initial study to evaluate superficial lesions of the parotid glands and can provide a rapid and accurate diagnosis of lipoma. 15 16 On a sonogram, it is possible to recognize an elliptical mass parallel to the skin's surface that is hyperechoic in comparison to surrounding muscle and contains linear echogenic lines that are parallel to the ultrasound beam. 12 In an USG, we can find a hyperechoic mass that is adjacent to muscles and contains a linear, echogenic line at right angles to the beam. However, some may be hypoechoic or isoechoic; hence, it is less pathognomonic compared with other scans. 2 As in this case, the USG visualized solid mass in the left parotid with sharp and flat margins and a slightly hypoechoic mass in left parotid region. These results had to be confirmed with an MRI examination to ensure that the perioperative diagnosis was lipoma.

The MRI results showed a mass with the intensity of fat growing on the left parotid region. An MRI can be useful in providing details about a tumor's clinical characteristics and in identifying the tumor's location and relationship to significant anatomical structures. Higher soft tissue contrast, enhanced lesion definition, and the absence of ionizing radiation are some of the benefits of an MRI. 16 By comparing the signal strength on T1- and T2-weighted images, an MRI is still the best tool for accurately diagnosing lipomas prior to surgery. 9 14 16 With a higher signal intensity on T1- and T2-weighted images without contrast media enhancement and a weak signal on fat-suppressed images, an MRI reveals the typical signal intensity patterns. 14 15 With a “black rim” surrounding the tumor, it may also identify clearly between lipoma and normal adipose tissue (subcutaneous tissue). 1 9 Furthermore, MRIs are precise, safe, and incur little biological expenses for the patient, even though their price is higher compared with computed tomography. The MRI was the best diagnostic technique for determining the characteristics of the tumor margin and the involvement of the surrounding tissues since it supplied all the information needed for either diagnosis or preoperative planning. 3 8 15

Deep parotid lobe lipomas are highly uncommon and difficult to treat; this requires meticulous dissection of the facial nerve's branches. 1 17 Different surgical methods are used based on the topography, tumor size, pathology, and relationship to the surrounding areas. In the case of parapharyngeal extension, a near complete parotidectomy is performed in addition to a superficial parotidectomy, extracapsular dissection, partial excision of the inferior part of the parotid gland, and superficial parotidectomy. 1 3 Most surgeons advise superficial parotidectomy with facial nerve dissection prior to the excision of lesions in the deep lobe during surgery. 1 14 In this case, we underwent a superficial parotidectomy and used blunt dissection to expose all of the facial nerve's branches from its main trunk. The technique minimized the incidence and recurrence rate of postoperative Frey's syndrome. 8 18 As we mentioned in this case, the lipoma was located on the main trunk and its peripheral branches, and a Blair approach was used to remove the lesion. The Blair incision's benefits are complete gland exposure and great access to the facial nerve. Furthermore, it is esthetically pleasing and concealable when positioned in a natural skin crease. 19

Facial nerve dysfunction, Frey's syndrome, facial scars, or an asymmetrical contour are a few potential postoperative morbidities that could arise and should be discussed with the patient. 8 18 The primary concerns should be the postoperative esthetic and functional outcomes. Following surgery for benign tumors of the parotid gland, facial nerve injury ranged from 8.2 to 65%. Therefore, after a whole parotidectomy was done to treat a deep lobe parotid lipoma, a high rate of facial nerve impairment has been reported. 1 17 Facial nerve function is maintained by careful dissection that exposes all of the nerves in full, as well as by the use of a facial nerve monitor. 1 14 The patient felt that the lump on the left cheek had disappeared, and postoperative symmetric face contour had returned after surgery. There was no difficulty in moving the lips when smiling and moving the eyelids and lips after surgery. The patient felt that the postoperative wound healing was uneventful with no complications and no facial scars that interfered with her appearance after surgery.

It is preferable not to postpone the surgery for too long, as a local mass effect may be complicated with facial nerve palsy or even airway obstruction if parapharyngeal space is involved. 2 When the tumor is well capsulated after surgery, the recurrence probability for lipomas is 5%. 9 10 18 The preferred surgery approach is a superficial parotidectomy with facial nerve preservation, as it allows a better clearance of the tumor with less risk of recurrence. 2

In this case, excision of the lipoma and superficial parotidectomy were performed with appropriate facial nerve dissection so that the entire lipoma could be excised without injuring the facial nerve. This is important to prevent postoperative facial nerve dysfunction. However, this case has some limitations, follow-up of the patient was affected by the coronavirus disease 2019 pandemic, so patient evaluation could not be routinely conducted. Postoperative evaluation was done by clinical examination rather than postoperative MRI, as the tumor was well-capsulated with a low risk of recurrence, and a superficial parotidectomy was performed to ensure that no tumor tissue remains.

## Conclusion

Lipoma of the parotid gland is uncommon and can be clinically deceptive. An accurate clinical assessment is required, followed by preoperative imaging and a histopathological examination to facilitate in the diagnosis of parotid gland lipoma. For the histopathological examination, an FNAB is highly accurate for the diagnosis of lipomatous tumors. For the purpose of facilitating the identification of lipoma, preoperative pictures from a USG and MRI are highly useful in evaluating its location and its relationship to significant anatomic structures. The best tool for both diagnosis and surgical planning remains to be an MRI.

Due to the possibility of a facial nerve lesion, surgical management of parotid region lipomas is difficult and should only be performed by skilled surgeons. Postoperative functional and esthetic outcomes should be the main objectives, and in all cases, a superficial parotidectomy and blunt dissection of facial nerve trunks are required.
